# Regulated Phase Separation in Al–Ti–Cu–Co Alloys through Spark Plasma Sintering Process

**DOI:** 10.3390/ma17020304

**Published:** 2024-01-07

**Authors:** Seulgee Lee, Chayanaphat Chokradjaroen, Yasuyuki Sawada, Sungmin Yoon, Nagahiro Saito

**Affiliations:** 1 Department of Chemical Systems Engineering, Graduate School of Engineering, Nagoya University, Furo-cho, Chikusa-ku, Nagoya 464-8603, Japan; sg@sp.material.nagoya-u.ac.jp; 2 Department of International Collaborative Program in Sustainable Materials and Technology for Industries between Nagoya University and Chulalongkorn University, Graduate School of Engineering, Nagoya University, Furo-cho, Chikusa-ku, Nagoya 464-8603, Japan; eig@sp.material.nagoya-u.ac.jp; 3 Institute of Innovation for Future Society, Nagoya University, Nagoya 464-8601, Japan; ysawada@sp.material.nagoya-u.ac.jp; 4 Department of Micro-Nano Mechanical Science and Engineering, Nagoya University, Furo-cho, Chikusa-ku, Nagoya 464-8603, Japan; yoon.sungmin.v8@f.mail.nagoya-u.ac.jp; 5 Conjoint Research Laboratory in Nagoya University, Shinshu University, Furo-cho, Chikusa-ku, Nagoya 464-8603, Japan; 6 Japan Science and Technology Corporation (JST), Open Innovation Platform with Enterprises, Research Institute and Academia (OPERA), Furo-cho, Chikusa-ku, Nagoya 464-8603, Japan

**Keywords:** Al-Ti-containing multicomponent alloys, phase separation, hardness, spark plasma sintering, powder metallurgy

## Abstract

With the goal of developing lightweight Al-Ti-containing multicomponent alloys with excellent mechanical strength, an Al–Ti–Cu–Co alloy with a phase-separated microstructure was prepared. The granulometry of metal particles was reduced using planetary ball milling. The particle size of the metal powders decreased as the ball milling time increased from 5, 7, to 15 h (i.e., 6.6 ± 6.4, 5.1 ± 4.3, and 3.2 ± 2.1 μm, respectively). The reduction in particle size and the dispersion of metal powders promoted enhanced diffusion during the spark plasma sintering process. This led to the micro-phase separation of the (Cu, Co)_2_AlTi (L2_1_) phase, and the formation of a Cu-rich phase with embedded nanoscale Ti-rich (B2) precipitates. The Al–Ti–Cu–Co alloys prepared using powder metallurgy through the spark plasma sintering exhibited different hardnesses of 684, 710, and 791 HV, respectively, while maintaining a relatively low density of 5.8–5.9 g/cm^3^ (<6 g/cm^3^). The mechanical properties were improved due to a decrease in particle size achieved through increased ball milling time, leading to a finer grain size. The L2_1_ phase, consisting of (Cu, Co)_2_AlTi, is the site of basic hardness performance, and the Cu-rich phase is the mechanical buffer layer between the L2_1_ and B2 phases. The finer network structure of the Cu-rich phase also suppresses brittle fracture.

## 1. Introduction

A lightweight design for components, such as reducing the weight of parts in automobiles and aircraft, can largely improve energy efficiency and reduce CO_2_ emission. Al and Ti alloys have been widely used, due to their lightweight and ease of processing [1]. However, the improvement in their mechanical properties is still ongoing, aiming to expand their application area. Recently, the design of Al-Ti-containing multicomponent alloys to improve the properties of lightweight materials has been proposed. For example, Huang et al. reported that the AlCrTiV alloy exhibited a hardness of 710 HV with a density of 4.5 g/cm^3^ [2]. Additionally, Liao et al. investigated a series of Ti_x_(AlCrNb)_100−x_ alloys with hardness values of 290–516 HV and a density of 4.85–5.25 g/cm^3^ [3]. X–Al–Ti alloy systems also have been developed to improve mechanical properties through ordered body-centered cubic (BCC) (B2) phase-strengthened alloy [4,5]. While previous studies have emphasized the high hardness of the B2 phase, it is imperative to recognize that the presence of the B2 phase within the metallurgical structure does not universally enhance the material’s mechanical properties. To solve this problem, there has been growing attention on the examination of the L2_1_ phase as another ordered BCC phase. This phase is characterized by a higher degree of ordering than the BCC and B2 phases, following the sequence: BCC < B2 < L2_1_ [6]. Specifically, the L2_1_ phase exhibits a restricted slip system and a better resistance to creep than the B2 and BCC phases [7]. Consequently, L2_1_ has been proposed to improve strength and ductility significantly [8]. In the Co–Al–Ti systems, the L2_1_ and B2 phases are known to exhibit a phase-separated structure [9], and Cu stabilizes the L2_1_ phase [10]. Therefore, in this study, the addition of Cu into the Co–Al–Ti system was anticipated to elevate the ratio of the L2_1_ phase and enhance the mechanical properties. Moreover, concerning weight density, the suitable range for lightweight materials is below 7.00 g/cm^3^ [11,12]. In the case of the X–Al–Ti (X = Cu, Co) system, the weight density is approximately 6.8 g/cm^3^, so the addition of Cu does not compromise lightweight characteristics.

Various methods are used to fabricate alloys, such as arc melting [13], mechanical alloying [14], and hot isostatic pressing [15]. Among these methods, powder metallurgy based on spark plasma sintering (SPS) has advantages due to its rapid heating and cooling process. Compared to conventional sintering, SPS is recognized for producing nanocrystalline alloys through high densification achieved in a short duration [16]. The heat treatment through SPS enables a material design that effectively preserves local structures, referred to as non-equilibrium states, making it an excellent process for optimizing metal microstructures and achieving nanoscale crystallization. In addition, the morphology of powder, particularly its dimension, could significantly influence the final microstructure in powder metallurgy using SPS. A reduced particle size generally results in finer grain size, contributing to the enhancement of mechanical properties [17]. Another aspect of improving these mechanical properties involves optimizing phase-separated microstructures. For optimized phase-separated microstructures, it is necessary to implement a non-equilibrium process in which no bonding occurs between the phase regions.

Considering the aforementioned perspective, this study systematically varied the particle size of Al, Ti, Co, and Cu powder mixtures through the powder metallurgy process with SPS to optimize the phase-separated microstructure featuring the refinement of L2_1_ and its effect on hardness was investigated. Planetary ball milling was conducted under three duration conditions: 5, 7, and 15 h, as a process parameter for particle size reduction. The alloys were then fabricated through SPS, utilizing metal powders prepared by ball milling. The resulting samples were carefully evaluated using scanning electron microscopy (SEM), energy dispersive spectroscopy (EDS), X-ray diffractometry (XRD), and high-resolution transmission electron microscopy (HRTEM). Hardness measurements were carried out using a Vickers hardness tester. Finally, the correlation between the phase-separated microstructure and the resultant mechanical properties was elucidated.

## 2. Materials and Methods

The initial powders of Al (<30 µm), Ti (<44 µm), Cu (<100 µm), and Co (<43 µm) (The Nilaco Co., Ltd., Tokyo, Japan) with high purity (>99.5 wt.%) were used, and stearic acid was added as the process control agent (PCA) for lubrication. Each elemental powder with 25 at. % was used and mechanically milled using a high-energy planetary ball mill (Pulverisette 7, Fritsch Japan Co. Ltd., Tokyo, Japan) with 5 mm diameter zirconia balls and zirconia vials (45 mL) under a high-purity argon atmosphere. The milling times were 5, 7, and 15 h, respectively. In addition, 0.5 wt.% of PCA was added during the 7 h of milling, followed by an additional 0.5 wt.% PCA for continuous milling up to 15 h. The ball-to-powder ratio was 10:1, and the rotational speed was 300 rpm. The ball milling was stopped for 10 min every 30 min to prevent overheating. The ball-milled powder mixtures were compacted under vacuum conditions (about 5 Pa) using SPS equipment (SPS-211LX, Fuji Electronics Industry Co., Osaka, Japan). The hypothetical melting point of the alloy ($T_{m}$) is calculated as follows:

(1) $$T_{m}=\sum_{i=1}^{n}c_{i}{\left(T_{m}\right)}_{i}$$

where $c_{i}$ is the atomic percentage of the *i*th component element, and ${\left(T_{m}\right)}_{i}$ is the melting point of the *i*th component element. The T_m_ of Al–Ti–Cu–Co alloy is 1227 °C, and the necessary sintering temperature level is generally between 0.6 and 0.8 T_m_ [18]. Considering this, along with our preliminary experiments, we set the optimal sintering temperature at 900 °C. During the sintering process, a pressure of 30 MPa was applied, with a holding time of 3 min, as illustrated in the heating/cooling profile provided in Figure S1. The obtained cylindrical samples with approximately 10 mm diameter and 2 mm thickness were polished with SiC paper from 320 to 2000 grit, followed by final mirror polishing using 1 µm diamond powder. The samples obtained in this study were classified according to the milling time; samples sintered at 900 °C using powders for 5, 7, and 15 h ball-milled powders were designated as 5 h–900, 7 h–900, and 15 h–900, respectively.

The unmilled and ball-milled powder mixtures and sintered samples were characterized using an XRD (Smartlab, Rigaku Co., Tokyo, Japan) equipped with monochromatic Cu Kα radiation (λ = 1.5418 Å) X-rays source with scanning at 2θ angle from 20 to 100 degrees at a scan rate of 3 deg/min. Subsequently, the microstructures were examined using SEM (S-4800, Hitachi, Tokyo, Japan) equipped with EDS at an accelerating voltage of 15 kV. Electron backscatter diffraction (EBSD) analysis was also conducted to reveal the phase orientation of the sintered sample using a cold-field emission SEM (SU-8230, Hitachi, Tokyo, Japan) equipped with an HR EBSD detector. Before EBSD scanning, the final polishing of the samples was conducted using an ion milling system (IM4000, Hitachi, Tokyo, Japan). EBSD detector interfaced with the scan region of 100 μm × 150 μm and scan step of 0.5 μm. TEM specimens were prepared using a focused ion beam (FIB, FB-2100, Hitachi, Tokyo, Japan) for cross-section, and then analyzed with HRTEM (JEM-2100, JEOL Ltd., Tokyo, Japan) and field emission SEM (FE-SEM, JSM-7610 F, JEOL Ltd., Tokyo, Japan) at an accelerating voltage of 200 kV. The average particle size distribution was assessed based on SEM images (Figure S2) using ImageJ software (version 1.54d, National Institutes of Health, Bethesda, MD, USA) at least 800 spots for each sample. The density of the sintered sample was measured by an electro-densimeter (MDS300, Alfa Mirage Co., Ltd., Osaka, Japan) based on Archimedes’ principle and automatically calculated from sample weights in air and water. Vickers hardness values were obtained on the polished surface of the samples using a Vickers hardness tester (FM-700, Future-tech Co., Tokyo, Japan). The applied load was 1 kgf with a dwell time of 15 s. The hardness test was carried out 10 times on the sample, and then the average of the resulting values was determined.

## 3. Results and Discussion

### 3.1. Characterization of Ball-Milled Powders

**Figure 1:**
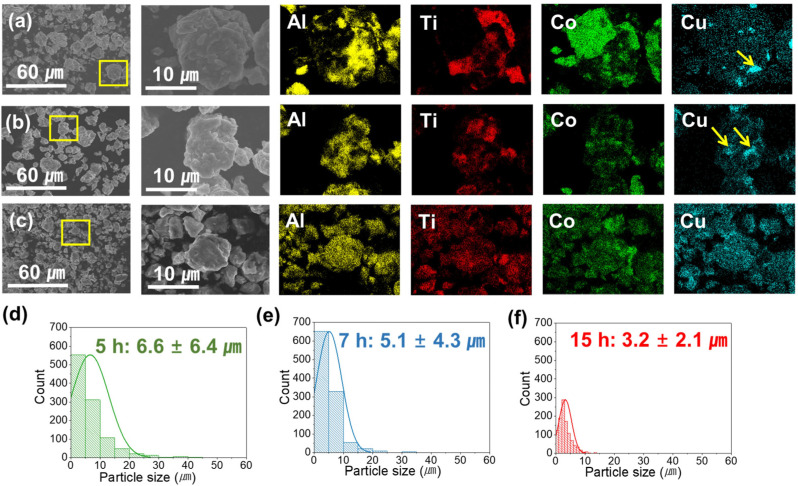
SEM/EDS results of the ball-milled Al–Ti–Cu–Co powders at (**a**) 5 h, (**b**) 7 h, and (**c**) 15 h, each yellow box in (**a**–**c**) indicates the magnified region on the right SEM image, respectively and the particle size distribution of the ball-milled Al–Ti–Cu–Co powders at (**d**) 5 h, (**e**) 7 h, and (**f**) 15 h.

The morphology, alloying tendency, and average particle size of ball-milled powders were confirmed with SEM and EDS (Figure 1). In Figure 1a, 5 h ball-milled powders exhibited a partial alloying of ductile Al and Cu with copper segregation. Additionally, Ti and Co were not alloyed with other elements and were unevenly mixed. With the extension of ball milling time at 7 and 15 h (Figure 1b,c), the particle size reduction and alloying further progressed, leading to the homogeneous distribution of Ti and Co. Finally, the 15 h ball-milled particles showed uniform mixing with the finest particle size. Moreover, some degree of copper segregation observed at 5 and 7 h (arrow mark) became more refined and evenly distributed at 15 h. As depicted in Figure 1d–f, the average particle sizes of powders subjected to 5, 7, and 15 h of ball milling were 6.6 ± 6.4, 5.1 ± 4.3, and 3.2 ± 2.1 μm, respectively. Both the average particle size and size deviation exhibited a decrease with increasing ball milling time. In this study, ball milling was terminated at 15 h, when it was confirmed that equiaxed morphology with fine size was consistently achieved. The extension of ball milling time can lead to the intense cold welding and agglomeration of powders. Normally, an additional PCA is required to prevent these phenomena; however, the addition of PCA could influence the contamination level of final products [19]. For example, Anas et al. reported that PCA promoted powder oxidation and subsequently increased oxide contamination in bulk samples [20]. Furthermore, prolonged ball milling time could increase impurities from the ball milling medium (e.g., container and ball) [21].

**Figure 2:**
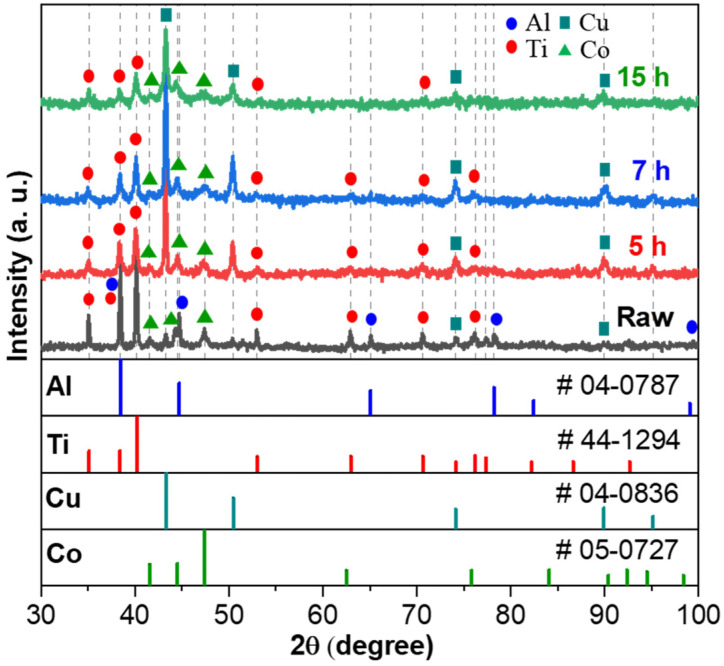
XRD pattern of unmilled Al–Ti–Cu–Co powders, and ball-milled Al–Ti–Cu–Co powders at different ball milling times at 5, 7, and 15 h (#: powder diffraction files (PDF) card no.).

The XRD pattern of the unmilled and the ball-milled powders after 5, 7, and 15 h are shown in Figure 2. The diffraction peaks of unmilled powders clearly showed the pattern attributed to pure elemental powder. The disappearance of diffraction peaks attributed to Al after 5 h of ball milling indicates that Al alloyed with other elements (Ti, Co, Cu). As the ball milling time at 5 and 7 h was prolonged, the decrease and broadening of the peaks corresponding to Ti and Co were observed, referring to a severe lattice distortion and grain size reduction induced by the high-impact energy generated during planetary ball milling [22]. Finally, the primary FCC peaks attributed to Cu and the minor HCP peaks corresponding to Co and Ti were observed at 15 h ball-milled powders. Additionally, the reduction in the overall peak intensity occurred, implying that a severe plastic deformation was induced by the ball milling process that contributed to fine grain sizes and increased internal stresses [23]. The primary objective at this stage is the reduction in particle size and the uniform mixing of powders, and the observed alloying reactions did not significantly impact the subsequent process.

### 3.2. Phase-Separated Microstructure of the Al-Ti-Cu-Co Alloys Obtained with SPS

**Figure 3:**
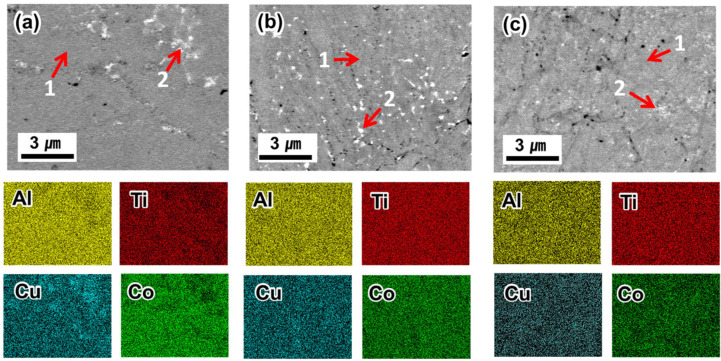
BSE images with EDS mapping of (**a**) 5 h–900, (**b**) 7 h–900, and (**c**) 15 h–900.

Figure 3 shows the backscattered electron (BSE) image with EDS mapping of the Al–Ti–Cu–Co alloys (designated as 5 h–900, 7 h–900, and 15 h–900, respectively), which were sintered at 900 °C using ball-milled powders with durations of 5, 7, and 15 h. The BSE mode provided composition contrast based on atomic number, which was used to distinguish different phases in the obtained Al–Ti–Cu–Co alloys. EDS results for each phase representing the different contrasts observed in this alloy are presented in Table 1. Along with the EDS mapping and elemental composition, it could be suggested that the predominant phase corresponding to the dark gray contrast (mark 1) represented the Al–Ti–Cu–Co phase region with similar atomic ratios corresponding to the (Cu, Co)_2_AlTi phase. The precipitated phase with white contrast (mark 2) had a smaller volume fraction and corresponded to a Cu-rich phase. The coarse Cu-rich phase became finer and more homogeneously distributed with increasing milling time, as shown in Figure 3a–c. The EDS composition results also revealed the homogenization of Cu within the predominant (Cu, Co)_2_AlTi phase as milling time was prolonged, i.e., 5 h (24.4 at.%) < 7 h (27.4 at.%) < 15 h (28.4 at.%). According to the previous results of Figure 1, the segregated Cu in the ball-milled powders at 5 and 7 h could affect the segregation of the Cu-rich phase during sintering. Furthermore, a finer and more uniform powder distribution without Cu segregation was finally obtained when the milling time was increased. This affected the homogeneous distribution of nano-sized Cu-rich precipitates during the SPS process. 

In addition, a higher concentration of Al in the Cu-rich phase was observed in EDS point analysis, indicating preferential Cu-Al bonding. Additionally, the presence of Cu in the (Cu, Co)_2_AlTi phase suggests that Cu is also actively combined with other elements. This could be explained by the dominant role of diffusion rate during the sintering process. In general, the diffusion rate is closely related to the melting point of the elements. Elements with lower melting points exhibit weaker atomic bonds and higher diffusion coefficients [24]. The alloying sequences of the Al–Ti–Cu–Co systems could be Al → Cu → Co → Ti; therefore, Al and Cu preferentially diffuse and combine. During sintering, the molten Al (Al(*l*)) covers the surface of solid Cu (Cu(*s*)), initiating a chemical reaction on the Cu surface, ultimately leading to the formation of a Cu-rich phase. Subsequently, the remaining Al rapidly combined with other elements to form (Cu, Co)_2_AlTi phase. Finally, Cu in the Cu-rich phase diffused and moved into the (Cu, Co)_2_AlTi phase, leading to a decrease in the fraction of the Cu-rich phase and an increase in the Cu content of the (Cu, Co)_2_AlTi phase.

**Figure 4:**
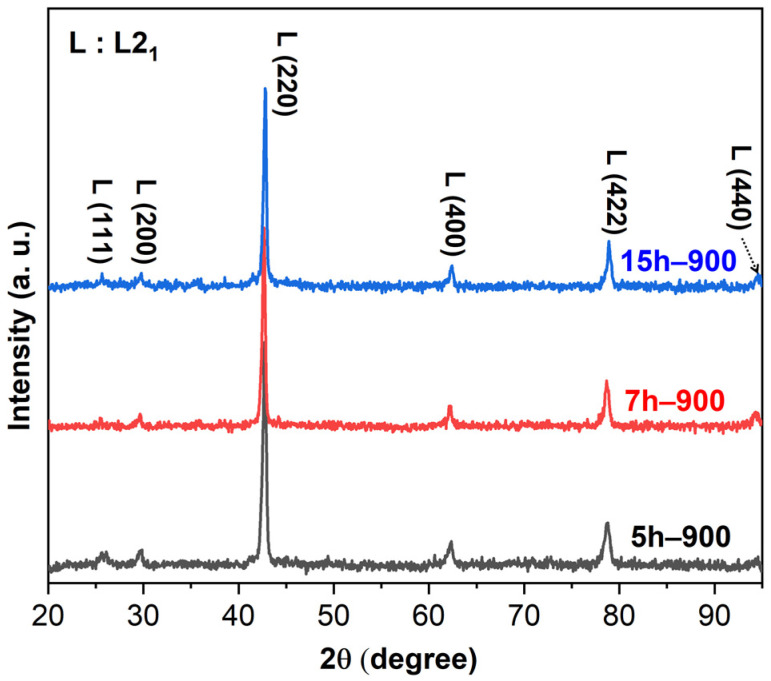
XRD results of the Al–Ti–Cu–Co alloys corresponding to 5 h–900, 7 h–900, and 15 h–900.

Figure 4 shows the XRD patterns of the 5 h–900, 7 h–900, and 15 h–900 samples. Compared to the XRD patterns of the ball-milled powder, it showed that phase transformation occurred after the SPS. The microstructure of the Al–Ti–Cu–Co alloys dominantly exhibited diffraction peaks corresponding to the Heusler L2_1_ phase (AlCu_2_Mn prototype, Fm3m, cF16). The L2_1_ phase is a highly ordered BCC-based structure that consists of eight smaller BCC lattices [25]. The Cu-rich phase was not detected due to its very low volume fraction (<2 vol.%) and nanocrystalline property [26]. In multi-component alloys, the crystal structure can be significantly distorted because of the randomness with which different-sized atoms occupy lattice points. This intrinsic lattice distortion can increase the scattering effect, ultimately weakening the detectable diffraction signal [27]. Combined with BSE and EDS results, the dominant phase is (Cu, Co)_2_AlTi, represented by the dark gray phase with most of the volume fraction (Figure 3).

**Figure 5:**
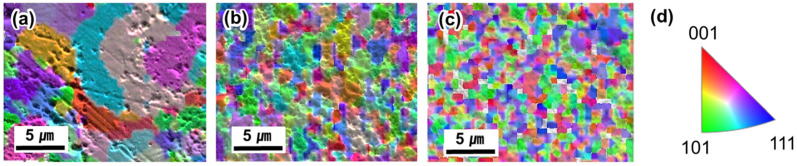
IPF orientation colored grain map overlaid with EBSD band contrast image of (**a**) 5 h–900, (**b**) 7 h–900, (**c**) 15 h–900, and (**d**) standard stereographic triangle.

Figure 5 depicts the inverse pole figure (IPF) of the 5 h–900, 7 h–900, and 15 h–900, respectively. The Al–Ti–Cu–Co alloy showed a random crystalline orientation. It is noteworthy that the grain size became finer from 3.1 ± 1.5 μm, 1.8 ± 1.0 μm to 1.0 ± 0.5 μm, depending on the increased ball milling time of the powders. The grain morphology exhibited a more equiaxed shape. The fine grain size achieved after ball milling significantly influenced the small grain size reduction by SPS; the SPS method is known for its rapid heating rate and short holding time, which promotes alloy densification and controlled grain growth [28]. The results suggested that the fine particle size induced by ball milling led to the fine grain size of the L2_1_ phase and the Cu-rich phase due to the fast heating and cooling rates of SPS.

**Figure 6:**
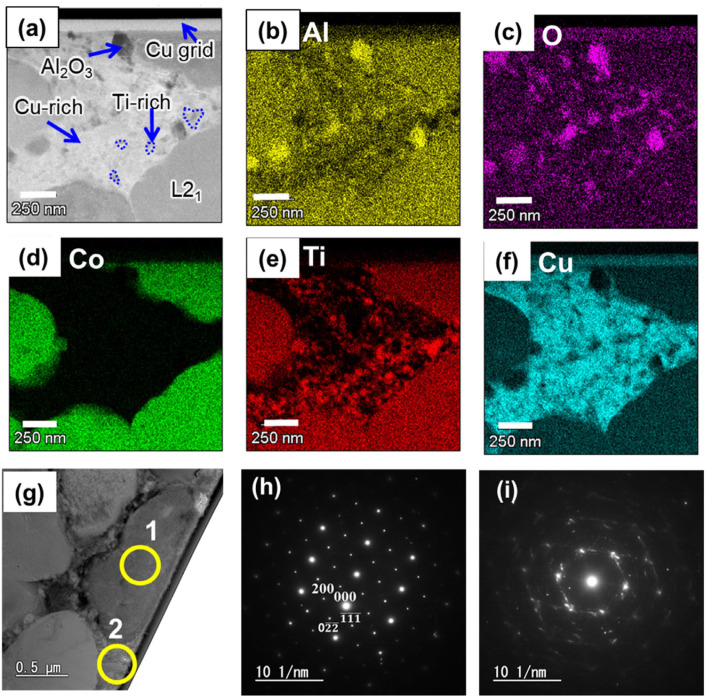
(**a**) TEM image of the 7 h-900, and TEM-EDS mapping elemental distribution of (**b**) Al, (**c**) O, (**d**) Co, (**e**) Ti, and (**f**) Cu, (**g**) bright-field TEM image, (**h**) diffraction pattern taken under the [110] zone axis from L2_1_ phase (marked 1 in (**g**)), and (**i**) diffraction pattern taken under the [111] zone axis from Cu-rich network phase (marked 2 in (**g**)).

L2_1_ and B2 are indistinguishable in XRD peaks because they have essentially the same crystal structure. Hence, the HRTEM/EDS analysis was performed on the cross-section of the 7 h–900 sample for the additional clarification of the phases. As depicted in Figure 6a–f, a nanoscale Ti-rich phase with a light gray contrast (marked in a blue circle) is distributed within the Cu-rich phase region with a white contrast. The dark spots on the sample indicate aluminum oxide inclusions produced by small amounts of oxygen adsorbed on the powder during the powder-handling process. Figure 6g–i depicts the ordered L2_1_ phase structure, and the Cu-rich phase revealed heterogeneous structures including precipitates. In the Al–Ti–Cu–Co alloy system, a phase separation into the B2 and L2_1_ phases has been previously reported at 1173 K (900 °C), and the presence of the B2 phase can be observed in the Ti-rich portion [29]. Therefore, in this study, the Ti-rich phase within the Cu-rich phase can be presumed to form the B2 phase. Consequently, HRTEM/EDS results further confirmed a phase-separated microstructure. Furthermore, the Cu-rich phase region was observed as a network phase that interconnects the L2_1_ and B2 phases.

### 3.3. Mechanical Properties

**Figure 7:**
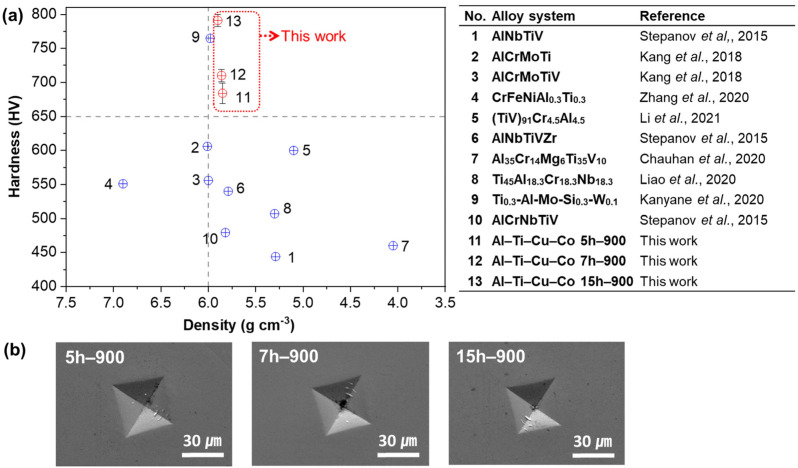
(**a**) Hardness and density graph of the Al–Ti–Cu–Co alloys compared to Al-Ti-containing multicomponent alloys [3,12,26,32,33,34,35,36,37]. (**b**) Indentation mark obtained after Vickers hardness test.

Figure 7a shows the hardness and density graph of the Al–Ti–Cu–Co alloys and the previously reported Al-Ti-containing multicomponent alloys as a comparative material. The hardness values of 5 h–900, 7 h–900, and 15 h–900 samples were 684 ± 15, 710 ± 9, and 791 ± 9 HV, respectively, which increased with prolonged ball milling time. Especially, a notable sharp rise was observed in the 15 h–900 sample. The Al–Ti–Cu–Co alloys exhibited a prominent combination of density and hardness compared to previously reported Al-Ti-containing multicomponent alloys. In addition, the indentation mark was distinguishable and had no cracking (Figure 7b), implying that the material could absorb enough energy to arrest cracking without embrittlement [30]. Furthermore, Table 2 presents the calculated strength and measured density of the Al–Ti–Cu–Co alloys compared with conventional metallic alloys. The Al–Ti–Cu–Co alloys exhibited relatively high specific strength (419–489 MPa cm^3^g^−1^) compared to conventional materials, such as Inconel 718 (1050 MPa cm^3^g^−1^) [10] and ultra-high tensile strength steel (1500 MPa cm^3^g^−1^) [31]. Finally, the Al–Ti–Cu–Co alloys could be considered promising candidate materials for advanced applications that are required for lightweight and high performance.

The strengthening mechanism of the Al–Ti–Cu–Co alloys, which exhibit excellent specific strength, can be explained as follows. Firstly, the high hardness of the Al–Ti–Cu–Co alloys is attributed to the presence of the L2_1_ phase. Zhang et al. reported that the BCC and ordered BCC phases in Al-Ti-containing multicomponent alloys increase both hardness and strength [33]. As a result, the formation of the highly ordered L2_1_ phase as the major phase contributed significantly to the high hardness of Al–Ti–Cu–Co alloys. Secondly, the particle size of the starting powder decreased as the ball milling time increased, resulting in a finer phase-separated structure after SPS. The reduced particle size and increased lattice strain generated by ball milling can accelerate the short-distance diffusion of constituent elements during SPS by providing more interfaces for phase formation [38]. This resulted in grain size reduction and uniform dispersion of the L2_1_ and Cu-rich phases, as evidenced by the increase in Cu concentration in the L2_1_ phase due to the facilitated elemental diffusion during extended ball milling time. In addition, the facilitated short-distance diffusion during SPS can result in the formation of non-equilibrium phases [39]. In conventional sintering methods like HIP, slow and extended heating and cooling rates allow sufficient time for the material to attain a state of thermodynamic equilibrium, leading to the formation of equilibrium phases through long-distance diffusion. In contrast, the rapid heating and short-time sintering in SPS facilitated the formation of optimized non-equilibrium phases through localized heating of the powder surface. As a result, the regulation of non-equilibrium phases yields distinctive material properties not achievable through conventional sintering processes. Lastly, precipitation hardening was achieved through the formation of Cu-rich phases, along with the B2 Ti-rich nanoprecipitate phase. The nanoscale size and uniform distribution of precipitates played a crucial role in precipitation strengthening. Nano-precipitates have been reported to act as a strengthening phase, impeding dislocation movement and enhancing the hardness [40]. Previous studies have demonstrated that the presence of L2_1_ phases, coupled with other precipitates, contributes to the enhancement of mechanical properties in multi-component alloys [41]. Therefore, nano-precipitates could significantly enhance the strength of the material through the precipitation strengthening effect. Consequently, phase-separated microstructure consisting of the finer grain L2_1_ phase and homogeneous distribution of Cu-rich phase led to a notable improvement in hardness. Finally, the Cu-rich phase was reinforced with a nanoscale Ti-rich B2 phase and served as a network phase connecting the hard L2_1_ and B2 phases, thereby mitigating embrittlement.

## 4. Conclusions

In this study, the phase separation microstructure of the Al–Ti–Cu–Co alloy was regulated by powder metallurgy processes and SPS using metal powder with varying particle sizes. Efforts were made to optimize the phase separation microstructure of the Al–Ti–Cu–Co alloys, and it was shown that the hardness increased, and brittle property was also suppressed, albeit at the Vickers level. Microstructure control of the L2_1_ phase of (Cu, Co)_2_AlTi and the Cu-rich phase incorporating the nanoscale Ti-rich B2 phase was achieved by increasing the ball milling time. The grain size reduction after powder metallurgy processes using SPS resulted in an enhancement in hardness (i.e., the hardness values of 5 h–900, 7 h–900, and 15 h–900 were 684 ± 15, 710 ± 9, and 791 ± 9 HV, respectively). This microstructure optimization can be achieved due to the rapid temperature rise and drop rate of the SPS process, which is a spatially non-equilibrium process. The materials obtained in this study, the Al–Ti–Cu–Co alloys, showed a good combination of hardness and density properties of 684–791 HV and 5.8–5.9 g/cm^3^ (<6 g/cm^3^) compared to other Al-Ti-multicomponent alloys and conventional materials (e.g., Inconel 718 and ultra-high strength steels).

## Figures and Tables

**Table 1:** Chemical compositions of different phases in the 5 h–900, 7 h–900, and 15 h–900.

Sample	Region	Al (at. %)	Ti (at. %)	Co (at. %)	Cu (at. %)
5 h–900	Overall	23.45	21.85	23.69	31.01
	Dark gray (1)	22.99	25.24	27.36	24.41
	White (2) *	18.84	12.48	10.88	57.79
7 h–900	Overall	23.06	23.49	23.47	29.98
	Dark gray (1)	23.20	24.69	24.73	27.38
	White (2) *	20.42	14.16	16.03	49.38
15 h–900	Overall	23.19	23.39	23.93	29.48
	Dark gray (1)	22.77	23.22	25.57	28.44
	White (2) *	23.79	19.83	14.84	41.55

* Note: due to the limitations of EDS resolution, the Cu composition may be underestimated by the interaction volume of the nano-sized white phase (2) and dark gray phase (1).

**Table 2:** The density, hardness, and strength of Al–Ti–Cu–Co alloys, Inconel 718, and ultra-high tensile strength steel (HTSS).

Alloys	Measured Density (g cm^−3^)	Hardness (HV)	Strength (MPa) *	Specific Strength (MPa cm^3^g^−1^)
5 h–900	5.85 ± 0.02	684 ± 15	2451	419
7 h–900	5.86 ± 0.02	710 ± 9	2557	436
15 h–900	5.90 ± 0.02	791 ± 9	2887	489
Inconel 718 [12]	8.18	355	1050	128
HTSS [31]	7.90	540	1500	190

* Calculated using the relationship. HV ≈ 3$\sigma_{y}$.

## Data Availability

Data are contained within the article and supplementary materials.

## Supplementary Materials

The following supporting information can be downloaded at: https://www.mdpi.com/article/10.3390/ma17020304/s1. Figure S1: Heating and cooling profile during SPS. Figure S2: SEM images of ball-milled Al–Ti–Cu–Co powders at (a–c) 5 h, (d–f) 7 h, (g–i) 15 h.
