# Supplementary material for: Regulated Phase Separation in Al–Ti–Cu–Co Alloys through Spark Plasma Sintering Process
Source: Materials (Basel). 2024 Jan 7;17(2):304. doi: 10.3390/ma17020304 (PMC10821483; doi:10.3390/ma17020304)
Supplement: Supplementary file 1 [file materials-17-00304-s001.zip › materials-2799186-supplementary.pdf]

Supplementary Information

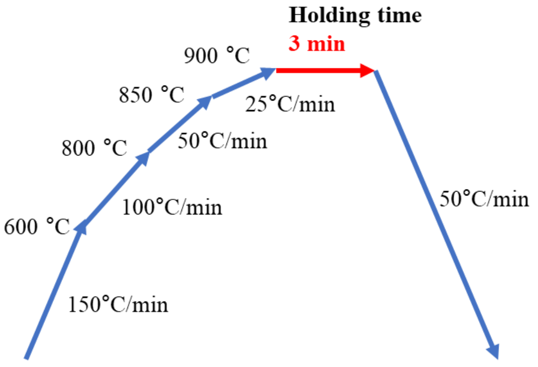

Figure S1 Heating and cooling profile during SPS**.**

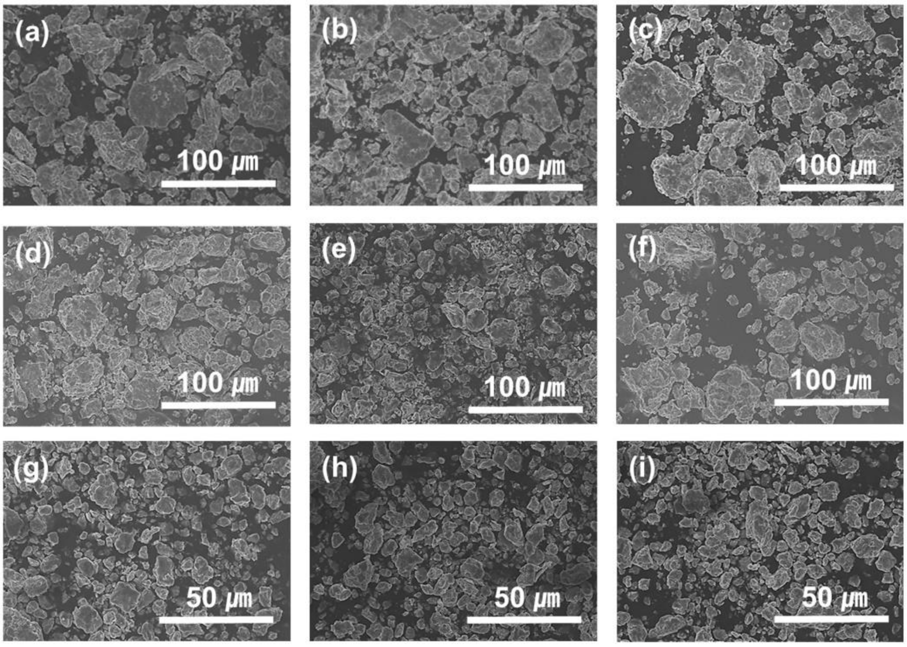

Figure S2 SEM images of ball-milled Al–Ti–Cu–Co powders at (a–c) 5 h, (d–f) 7 h, (g–i) 15 h.
